# A Unified Material Description for Light Induced Deformation in Azobenzene Polymers

**DOI:** 10.1038/srep14654

**Published:** 2015-10-06

**Authors:** Jonghoon Bin, William S. Oates

**Affiliations:** 1 Florida Center for Advanced Aero Propulsion (FCAAP), Department of Mechanical Engineering, Florida State University. Tallahassee, FL, 32310, USA

## Abstract

Complex light-matter interactions in azobenzene polymers have limited our understanding of how photoisomerization induces deformation as a function of the underlying polymer network and form of the light excitation. A unified modeling framework is formulated to advance the understanding of surface deformation and bulk deformation of polymer films that are controlled by linear or circularly polarized light or vortex beams. It is shown that dipole forces strongly respond to polarized light in contrast to higher order quadrupole forces that are often used to describe surface relief grating deformation through a field gradient constitutive law. The modeling results and comparisons with a broad range of photomechanical data in the literature suggest that the molecular structure of the azobenzene monomers dramatically influences the photostrictive behavior. The results provide important insight for designing azobenzene monomers within a polymer network to achieve enhanced photo-responsive deformation.

Azobenzene liquid crystals have well known light-matter coupling which have broad applications in adaptive optics^1^, energy harvesting^2^,^3^, artificial muscle^4^,^5^,^6^,^7^, and biology^8^. Whereas the photoisomerization process describing azobenzene molecular evolution has been studied extensively in the polymeric state^4^,^9^,^10^,^11^,^12^,^13^,^14^, the mechanisms linking light induced microstructure evolution to deformation in a polymer film are still debatable^15^,^16^,^17^,^18^. Similar complexities occur on other light-responsive polymers such as ones containing carbon nanotubes^19^. Light induced deformation in azobenzene liquid crystal polymer networks (azo-LCNs) can occur as bending and twisting of free standing films^7^,^12^ or from localized surface deformation (i.e., surface relief gratings (SRGs)^20^). Models have been developed to explain these different deformation processes^21^,^22^,^23^ but unifying the underlying photoisomerization process with macroscale photostriction from different light sources has been elusive. Insight on the photostrictive coupling illustrates how the polymer deformation may flip its sign as the molecular or quantum forces change as a function of the local interactions between the azobenzene and the polymer network^22^,^24^. We expand upon these arguments to illustrate how the same light induced microstructure reorientation of azobenzene gives rise to dramatically different photostrictive behavior within different polymer networks.

Azobenzene undergoes *trans-cis* photoisomerization from UV light (~365 nm) and a reverse *cis-trans* reaction when exposed to visible light (~450–500 nm) which affords unique polymer deformation control from polarized light excitation^6^,^25^,^26^,^27^. UV exposure results in a rod shaped azobenzene molecule with length approximately 10 Å transforming into a “kinked” shape with length of ~5.5 Å^27^; see Fig. 1. The higher energy *cis* state can be transformed back to its original rod shape (*trans* state) upon exposure to visible light or heat. Alternatively, exposure to blue-green light results in simultaneous *trans-cis* and *cis-trans* photochemical reactions due to the overlap in the optical absorption spectra. This process is known as *trans-cis-trans* photoisomerization. If the light is polarized, it can lead to what is known as the Weigert effect where the molecules in the *trans* state reorient to a plane orthogonal to the polarization direction^18^,^28^,^29^,^30^. When the azobenzene molecules are polymerized (pendent or cross-linked), free standing polymer films undergo significant bending or twisting that can be controlled by the polarization orientation of the light source^4^,^6^,^7^,^13^,^31^. Given the large shape anisotropy in the *trans* state, it is often assumed that azo-LCNs exhibit prolate behavior which predicts the majority of polarized induced free bending and twisting behavior^7^,^32^,^33^; however, direct extensions of such behavior to surface relief deformation has not been possible.

Deformation of azobenzene surface relief grating structures has been characterized by a mass diffusion process where field gradients associated with the spatial variation in intensity of the light beam gives rise to a surface shape change^15^,^20^,^34^. Whereas this modeling framework does not directly consider the material microstructure, it matches data considerably well for Gaussian laser beams with both linear and circularly polarized light^20^. More recently, these concepts have been extended to optical vortex beams which produce more complex surface texture that depends on the number of topological charges associated with the vortex beam^15^. In the vortex beam case, additional phenomenological coupling terms associated with optical absorption are introduced to simulate the polymer surface shape change via similar mass diffusion relations coupled to quasi-static linear momentum. These models are predicated on the use of a field gradient driving force (work conjugate to the quadrupole density) that is associated with the light beam to predict azobenzene polymer deformation. Such models will break down when simulating bending and twisting of films exposed to uniform light which contains no time-averaged field gradient on the material surface.

Here the implementation of the lower order dipole forces is shown to provide an explanation to the observed photomechanical deformation from uniform light exposure, linearly or circularly polarized light beams, and optical vortex beams. The theoretical framework relies on Maxwell’s time dependent electromagnetic equations, linear momentum equations, and equations associated with the electronic structure evolution of the azobenzene molecules due to light-matter interaction^35^,^36^. Dipole and higher order quadrupole forces are included in the electronic structure equations to assess their individual contributions and to compare against field-gradient mass diffusion models. The azobenzene and polymer network is homogenized over a microscopic representative volume element by treating the electronic coordinates of the azobenzene by two vector order parameters for the *trans* and the *cis* azobenzene states as shown in Fig. 1. As a result of this homogenization, the photostrictive coefficients are made dependent on the azo-monomer structure as motivated by experimental evidence^37^ and quantum/thermodynamic calculations^22^,^24^. It is shown that the spacers between the monomer and azobenzene pendent play an important role in photostrictive deformation.

## Results

### Theory

The set of equations used to describe azo-polymer photomechanical deformation is based on a Lagrangian density and a dissipative potential that contains free space energy, kinetic and stored energy of the solid, internal electronic structure, and dissipation due to photochemical reactions and light scattering. The conserved Lagrangian is described by 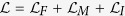 where *L*_*F*_ is the free space Lagrangian, 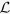
_*M*_ describes the kinetic and stored energy of the solid, and *L*_*I*_ describes light-matter interactions. Minimization of this Lagrangian leads to a fully conservative form of the time-dependent Maxwell’s equations, linear momentum, and a set of harmonic resonator equations governing the electronic structure and interactions with light^36^,^38^. Dissipation is included to quantify light absorption and scattering as described in subsequent paragraphs. An important component in this formulation is the light-matter Lagrangian, 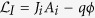, where *J*_*i*_ is the current density, *A*_*i*_ is the magnetic vector potential, *q* is the bound charge density, and *ϕ* is the electrostatic potential.

Optically active components within the charge density are defined to be a function of the internal state and the electric field. We invoke the dielectric assumption such that the total charge density is 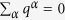 where *q*^*α*^ are local electron densities pertaining to relevant polymer or azobenzene constituents within the material. This summation to zero enforces electric neutrality. The effective charge densities of the *trans* and the *cis* state azobenzene are


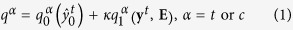


where 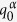 is a nominal charge density for the *trans* (*α* = *t*) and *cis* (*α* = *c*) states that accommodates changes to the local charge density that occur during *trans-cis* photoisomerization. It is defined as a function of the time-averaged magnitude of the electronic *trans* coordinate vector that is denoted by 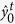. More details on the calculation of 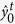 is given in the supplementary text. During *trans-cis-trans* photoisomerization, a state-dependent charge density, 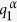, is introduced to simulate dichroic absorption and *trans-cis-trans* photoisomerization. This component is assumed to depend on the internal electronic *trans* vector and the electric field component, *E*_*j*_. A parameter *κ* is introduced to fully activate or deactivate the state-dependent charge density, which has the value of 0 and 1 for the *trans-cis* and *trans-cis-trans* photoisomerization, respectively. This is done to facilitate the simulation when quantifying each photoisomerization process separately. In general, *κ* = 1 such that *trans-cis-trans* processes are weighted by the dynamics of each electronic coordinate.

The state dependent charge density is described by the relations


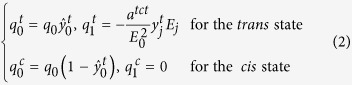


where *q*_0_ is a nominal charge density at zero field, *E*_0_ denotes the magnitude of the applied field and *a*^*tct*^ is a phenomenological parameter governing the amount of anisotropic absorption during *trans-cis-trans* photochemical reactions (see Table 1 in the supplementary material). In all discussion, we use indicial notation where *j* = 1, 2, 3^38^,^39^. The term 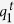 is important in describing reorientation of the *trans* state to a plane orthogonal to the electric field direction as described in subsequent paragraphs.

**Figure 1:**
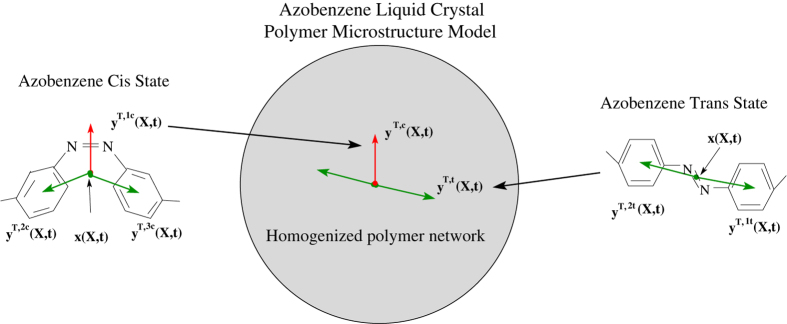
A description of the azobenzene liquid crystal polymer model used to simulate optically active microstructure and coupling with a homogenized polymer network.

The time-dependent electronic coordinates, 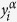, collectively describe optically active electrons within the azobenzene molecules that interact with light leading to molecular conformational changes and subsequent coupling with the polymer network. The representation of these electronic coordinates and a suitable reduced order set of microscale coordinates are shown in Fig. 1. We denote the effective electronic coordinates of each azobenzene state by the order parameters for the *trans* state as 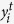 and for the *cis* state as 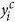. These coordinates are relative to the material’s center-of-mass, as denoted by **x**(**X**, *t*), such that all the momentum is carried by the mass center of the effective continuum element^38^,^39^.

It is known that azobenzene molecules are dichroic, which leads to preferred alignment in a plane orthogonal to the polarization of light^28^,^29^. This effect is governed by the state-dependent charge density term 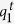 given by Eq. (2). The energy associated with the charge density is combined with a stored energy of the electronic *trans* state to illustrate driving forces during *trans-cis-trans* photoisomerization.

The stored energy of the electronic structure is defined to be a non-convex potential for both the *trans* and *cis* states. These stored energy density functions are


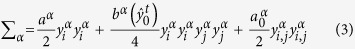


which includes separate stored energy functions for the *trans* (*α* = *t*) and *cis* (*α* = *c*) states. The phenomenological parameters, *a*^*α*^ and 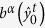, govern the evolution of the electronic coordinates and 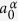 is a penalty on gradients of 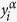 where 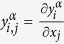. This term governs liquid crystal domain formation. A non-convex potential is produced by letting *a*^*α*^ < 0 and 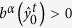. The higher order model parameter, *b*^*α*^, is defined to be a function of a time-averaged magnitude of the *trans* coordinate, 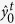, to model the slower time dynamics of photoisomerization relative to dynamics that occur at visible and UV light frequencies. The parameter 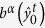 changes during *trans-cis* photoisomerization such that the *trans* coordinate reduces in magnitude while the *cis* coordinate increases from near zero to accommodate a loss of nematic order. The functional forms of these phenomenological parameters are assumed to be 
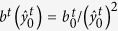
 and 
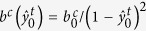
 where 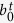 and 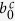 are positive constants. The time averaged electronic state is restricted to 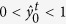 such that *b*^*t*^ and *b*^*c*^ are bounded. In the extreme limit, 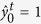 and 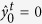 denote the fully *trans* and the fully *cis* state, respectively.

**Figure 2:**
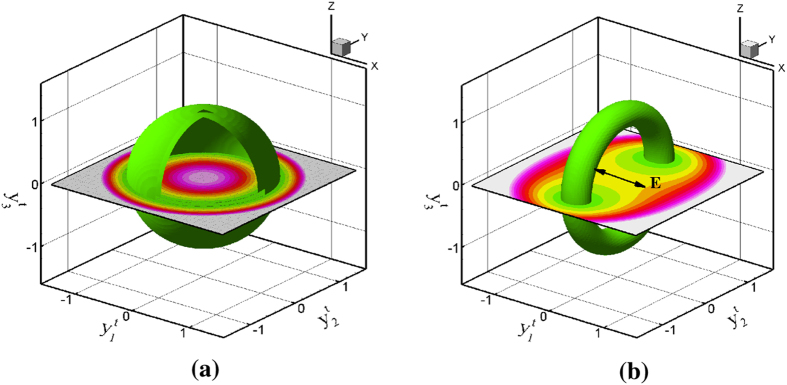
Iso-contours of the stored energy density for the *trans* state 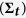 and the dipole interaction energy (*P*_*i*_*E*_*i*_) from 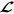
_*I*_, which is given by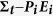. (**a**) Isotropic energy with no light present. (**b**) Energy surface when polarized light **E** is applied in the 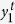 direction. The *trans* vectors tend to line up in a direction orthogonal to the light polarization as illustrated by the doughnut iso-energy surface.

The driving force for *trans-cis-trans* photoisomerization is illustrated in Fig. 2 for the low energy *trans* state prior to photoisomerization. This plot includes both the stored energy of the *trans* state from Eq. (3) and the electrostatic interaction energy of the *trans* charge density from Eq. (1); see the supplemental text for details. The electrostatic part of the Lagrangian interaction density is *qϕ* where *ϕ* is the electrostatic potential^38^. This energy density can be written in terms of the electric field and polarization as denoted by 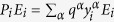. The energy plot illustrating the driving force for *trans-cis-trans* photoisomerization for the *trans* azobenzene coordinate is then 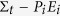. Figure 2(a) illustrates the zero light case where the *trans* state can orient into any direction; shown as an iso-energy, spherical surface in 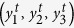 space. When linearly polarized light is aligned in the 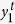 direction, for example, a driving force for the *trans* vector to reorient to any direction in the 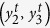 plane is created as shown by a doughnut of constant energy in these directions. The iso-energy doughnut in Fig. 2(b) contains this minimum energy radius on the 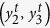 plane.

**Figure 3:**
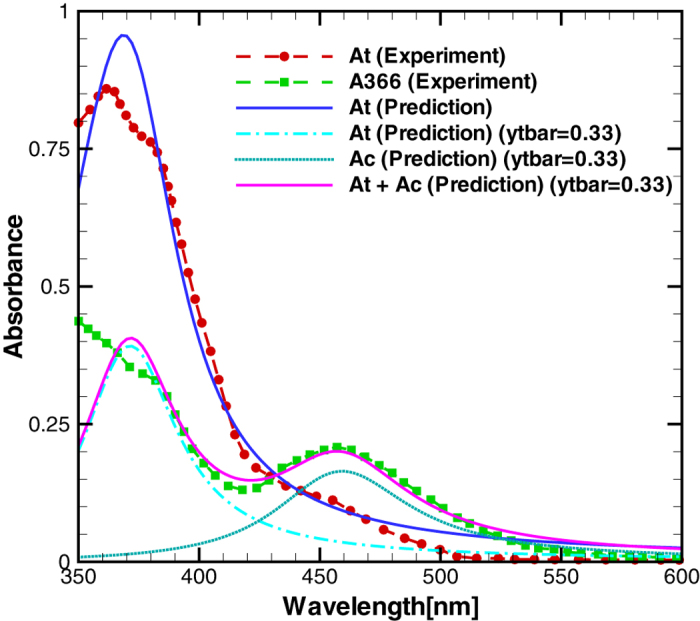
Comparison of absorption spectra of the azobenzene material between the numerical prediction described in the Results section and experiments^40^ for different *trans* and *cis* states. *At* denotes *trans* absorption and *Ac* denotes *cis* absorption.

A balance law based on the minimization of the Lagrangian energy density and internal dissipation describes the interaction of electronic structure with light and the polymer network; see the supplemental information for details. To quantitatively describe the nonlinear absorption characteristics of the azobenzene polymer, as shown in Fig. 3, a dissipation function D = 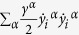 is used where losses are defined to be proportional to the rate of change of the optically active vector order parameters. In this relation, *γ*^*α*^ defines the amount of photochemical energy loss to heat and light scattering out of the material as opposed to light energy stored as photochemical energy in 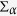 from Eq. (3). The electronic balance equation is obtained by minimizing the total Lagrangian density L and the dissipation function D with respect to the electronic coordinates 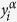. This results in


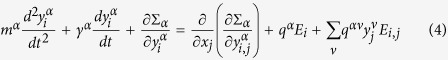


where we have neglected magnetic effects. The left hand side of this equation describes the electronic structure evolution which leads to the optical absorption shown in Fig. 3. All the parameters used are given in Table 1 in the supplementary material. The first term on the right hand side contains divergence of a microstress which governs liquid crystal domain structure formation. The last two terms on the right hand side describe forces associated with optically active charge density. The former term is the dipole force which is a function of the nominal charge density for a given electronic coordinate, *q*^*α*^, from Eq. (1) and the electric field, *E*_*i*_. The latter force is due to the quadrupole density which is a function of the charge density, *q*^*αv*^, the electronic coordinate, 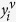, and the electric field gradient, *E*_*i,j*_^38^. This latter term is loosely analogous to the field gradient forces previously used to describe azo-LCN surface relief grating structures^15^,^20^. Through our numerical analysis, it was determined that the last term on the right hand side of Eq. (4) is negligible when simulating photomechanical deformation from uniform light sources and different types of polarized laser beams. Furthermore, if the dipole force is neglected, the shape change from polarized light does not follow experimental observations^15^,^20^.

For brevity, the additional balance equations required to minimize the total Lagrangian are not repeated here, but are included in the simulations and the supplementary discussion. They include the microscopic form of Maxwell’s equations and linear momentum. The key relation coupling electronic evolution of the azobenzene to the polymer network deformation is the Cauchy stress given by


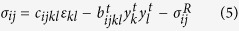


where we assume the deformation of the polymer to be quadratically proportional to the *trans* state of the azobenzene microstructure. A residual stress 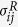 has been subtracted to account for the initial stress at equilibrium prior to light excitation. The phenomenological parameters include 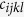 as the elastic tensor in which we assume to be isotropic^39^. The photostrictive tensor, 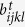, is more complex than the elastic tensor as it defines interactions between the polymer network and the *trans* azobenzene coordinates. The set of parameters used here is motivated by differences in the underlying azo-monomer structures.

It has been shown that the length of the spacers between a pendent azobenzene monomer (green rods in Fig. 1) and the polymer main chain significantly influence the photostrictive behavior of certain azobenzene polymer networks^37^. Changes in the length of the spacers between the azobenzene pendant and the main chain has been found to give opposite photostrictive behavior with respect to orientation of linear polarized light. Prior explanations associated with *trans-cis* photoisomerization under low intensity light include frictional forces and substrate clamping as a possible mechanism associated with the observed shape change^37^. Here we offer a different explanation that provides connections among a broader set of data. In the case of long spacers between the azobenzene pendant and main chain, the azobenzene reorients orthogonal to the polarized light during *trans-cis-trans* photoisomerization with a smaller change in the orientation of the polymer main chain. This assumes sufficient compliance afforded by the long spacer. Repulsive forces of the azobenzene lead to a contraction in the light polarization direction as a fraction of the azobenzene rods evolve to a plane orthogonal to the polarization direction. In the case where the spacers are short, stronger interactions between the main chain and the azobenzene are expected to occur leading to more alignment between the polarized light and the polymer main chain during azobenzene *trans-cis-trans* photoisomerization. In this case, the short spacer is assumed stiff and the azobenzene imparts larger forces on the main chain during photoisomerization. This process is expected to generate expansion in the direction of polarized light due to the main chain reorientation and contraction in orthogonal directions. These differences require the photostrictive tensor to be opposite in sign as a function of the azobenzene spacer length (see Table 2 in the supplementary material). Most surface relief grating experiments have been conducted with short spacers between a pendent azobenzene and the polymeric backbone^15^,^20^,^41^,^42^,^43^ while the majority of bending and twisting of free standing cantilever films contain azo-polymers with longer spacers in crosslinked azobenzene liquid-crystalline polymers^6^,^30^,^40^,^44^,^45^. It is also important to note the interactions between the *trans* and *cis* states during this process. In addition to *trans-cis-trans* photoisomerization, the model also accommodates *trans-cis* (order-disorder) photoisomerization and coupling with the polymer network. This behavior leads to model predictions of isotropic deformation on the volume average as the order of the *trans* state is reduced while the random *cis* state increases. The formation of the *cis* state will be compared to the reorientation of the *trans* state for circularly polarized beams and vortex beams to highlight how these two mechanisms contribute to photostriction. Based upon these arguments, we apply photostrictive parameters associated with the relevant azo-monomer structure and show how the proposed model predicts light induced deformation from different light sources and different film structures.

Due to the complexity of the azobenzene evolution and coupling between electromagnetics of light, polymer mechanics, and electronic structure excitation, we simulate the equations numerically using finite difference and finite element methods. Details of the procedure can be found in the Methods section.

### Numerical Analysis

The modeling response to uniform light is first summarized followed by simulations of circularly polarized laser beams and optical vortex beams. Surface relief grating validation for linear polarized light is included in the limiting case of zero topological charge in a vortex beam. The computations are based on the model geometry and boundary conditions described in subsequent sections and illustrated in the supplemental text. Polymer bending and microstructure evolution from linearly polarized light is described first for monodomain films. This is followed by analysis of surface texture evolution from different polarized light beams for polydomain films. We apply photostrictive coefficients for bending of cantilever films assuming long azobenzene spacers while in the case of surface relief deformation, we assume photostrictive coefficients corresponding to short azobenzene spacers. The parameter values are given in Table 2 in the supplemental text.

#### Uniform Light Induced Bending

**Figure 4:**
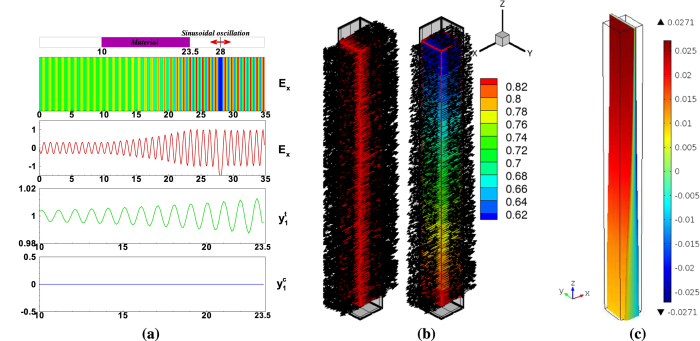
The material response to uniform light. (**a**) Plots of the polarized electric field component and the electronic material oscillation for the 100% *trans* state 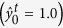. The one dimensional plots along the *z* direction, (0.5, 0.5, *z*), have been taken through the center line normal to the *xy* plane. Simulation was run with *λ* = 370 *nm*. (**b**) Azobenzene microstructure evolution of the *trans* state before and after light exposure. (**c**) Deformation due to light absorption and photoisomerization through the thickness where the color contour represents the displacement in the *x* direction. A rectangular parallelepiped of black lines represents the reference undeformed shape.

In this case, photomechanical *trans-cis* behavior of the azo-polymer is described in a monodomain film. Linearly polarized light in the *x* direction propagates in the *z* direction through the azo-polymer from a light source in the vacuum. Figure 4(a) represents the light and material response for the fully *trans* states (100% *trans* state) given previously in Fig. 3 when the polarized light source of wavelength *λ* = 370 *nm* (UV light) is applied. As shown in this figure, while the electric field waves propagate through the material, the azobenzene starts to absorb light energy. Once it reaches steady state oscillation, the magnitude of the electric field and the electronic displacement of the *trans* state decreases monotonically through the material’s thickness.

Figure 4(b) illustrates the distribution of the electronic coordinates at the initial and the steady state excitation states when the polarized light source is applied. The vectors are super-imposed on the time-averaged *trans* state 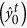 to illustrate the spatial variation through the material thickness. Experiments show that there is strong light absorption when the orientation of the *trans* coordinate vector is parallel to the applied electric field^30^. In this simulation, we assume that the *trans* state is initially aligned with the polarized light along the *x* direction to induce significant absorption. As the linearly polarized electric field propagates through the material, the azobenzene absorbs light energy leading to spatial variation through the thickness. In addition, the *cis* vector coordinates (not shown) start to increase from zero in a random pattern with the largest magnitude on the top and decreasing in magnitude through the thickness. The corresponding deformation, based on the stress given in Eq. (5), is shown in Fig. 4(c). The spatial variation of the concentration of the *trans* state 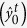 leads to a strain gradient in the *z* direction. The colors represent polymer displacements in the *x* direction highlighting that this volume element contracts near the surface exposed to light more than near the bottom. This would result in bending of a film towards the light source as typically observed experimentally for this particular microstructure and polarized light source. These results have also been validated for a polydomain where bending toward or away from the light is generated as the polarization is rotated 90°. More analysis of polydomain films is given for the case of surface relief deformation as follows.

#### Polarized Light Surface Texture Evolution

**Figure 5:**
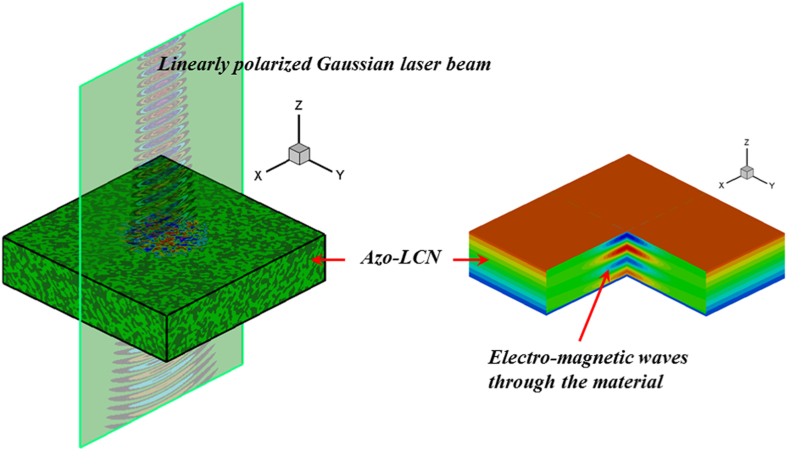
Description of a linearly polarized Gauss laser beam exposed to the material. Similar beam sources are used for the case of circular polarized beams and vortex beams.

We apply the same model to quantify complex surface texture changes from Gaussian beams circularly polarized and vortex beams with different topological charge. In the visible light regime, *trans-cis-trans* photoisomerization leads to more complicated evolution of the azobenzene microstructure as the *trans* state molecules reorient orthogonal to the polarized laser beam. In all simulations, the azo-polymer film is taken in a polydomain configuration as the initial reference state and exposed to a laser beam as illustrated in Fig. 5. The photostrictive coefficients assume short spacers between the azobenzene and main chain within the polymer network.

**Figure 6:**
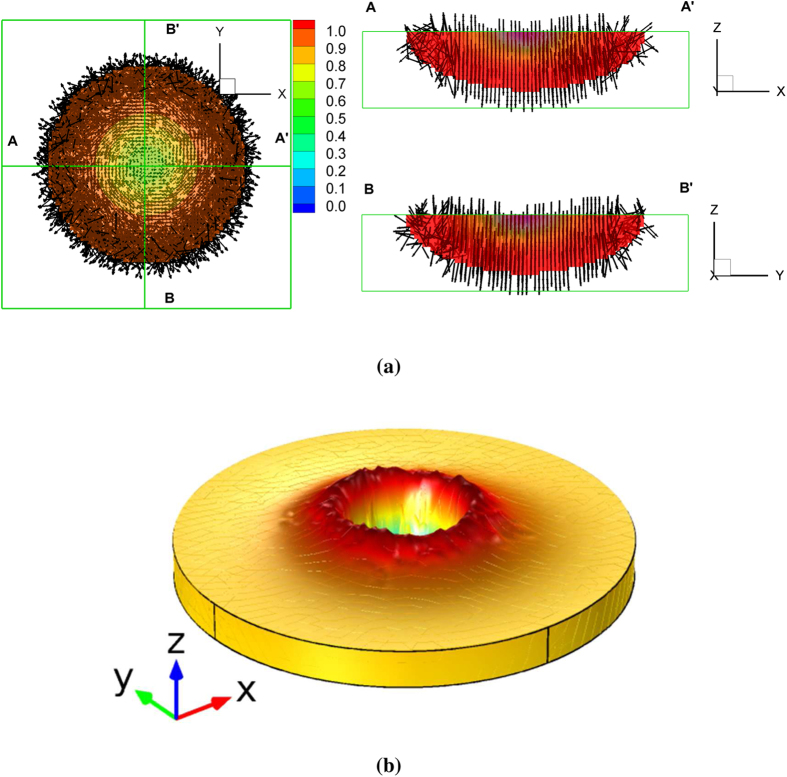
Numerical result in the case of a circularly polarized Gaussian laser beam. (**a**) The spatial distribution of the *trans* vector due to the light exposure (The color denotes the magnitude of the *trans* vector, 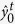. The region of 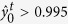 where the photoisomerization is negligible was filtered out). (**b**) Shape deformation (A color denotes the variation in the *z* direction).

Circularly polarized light is first considered where the electric field amplitudes *E*_1_ and *E*_2_ are equal and the phase angle is 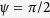. The electric field of the circularly polarized Gaussian laser beam given by 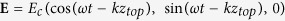 where 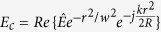 is applied on the top plane of the domain, *z* = *z*_*top*_. Figure 6 illustrates the spatial distribution of the *trans* vector due to the illumination of the circularly polarized Gaussian laser beam and the resultant deformation of the polymer. In a given *z* plane, the electric field vector rotates with constant angular velocity in the counter-clockwise direction. This field drives the *trans* state, initially randomly distributed, to reorient perpendicular to the electric field 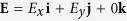. Since the field aligned in the (*x*, *y*) plane rotates on the femtosecond time scale, slower *trans-cis-trans* photoisomerization results in *trans* alignment predominantly in the *z* direction along the perimeter of the beam as shown in Fig. 6(a). More *trans-cis* photochemical reactions occur in the center reducing deformation due to the loss of order near the beam center. This is illustrated in Fig. 6(a) by the magnitude of the *trans* state shown by the color bar. In the supplemental text, the complementary formation of the *cis* state is shown further illustrating the order-disorder behavior near the center of the beam. Figure 6(b) illustrates the deformation attributed to the stress in Eq. (5). To minimize any effects from the boundaries, the medium of a cylindrical shape is considered for the computation of the mechanical deformation. On the bottom of the azobenzene polymer domain, the model is fully clamped, whereas traction-free boundary conditions are applied to the remaining boundaries. This figure shows that the photo-induced surface deformation is axisymmetric along the *z* axis. The protrusion along the outer rim is qualitatively in agreement with experiments given in the literature^20^. This illustrates that time-dependent fields coupled to optically active microstructure evolution and coupling with polymer deformation are also driven by the dipole forces in Eq. (4).

Surface deformation due to linearly polarized vortex laser beams with different topological charges is also studied. A Laguerre-Gauss (LG) beam is used for the light source which is denoted by 
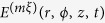
 in cylindrical coordinates where *m* and ξ represent the radial and the azimuthal mode index, respectively^46^,^47^. For 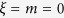, the solution reduces to a Gaussian polarized laser beam. For 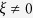, the LG mode has a vortex (or optical) phase governed by 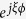. The optical phase of the higher-order LG mode varies by 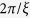, where ξ is called the vortex topological charge and can be a positive or negative integer. The phase at the vortex core (the *z* axis) is undefined and the optical field in this region vanishes as ξ increases, which finally leads to the doughnut shape of the optical intensity on the plane normal to the beam axis. The integer value of the vortex topological charge ξ determines the number of the helical structures of the wavefront and produces spatial variation of the optical intensity in the radial direction. The sign of ξ determines the handedness of a helical wavefront. A positive ξ leads to the spiral structure in the clockwise (or left-handed) direction, whereas a negative ξ defines the counter-clockwise (or right-handed) direction.

**Figure 7:**
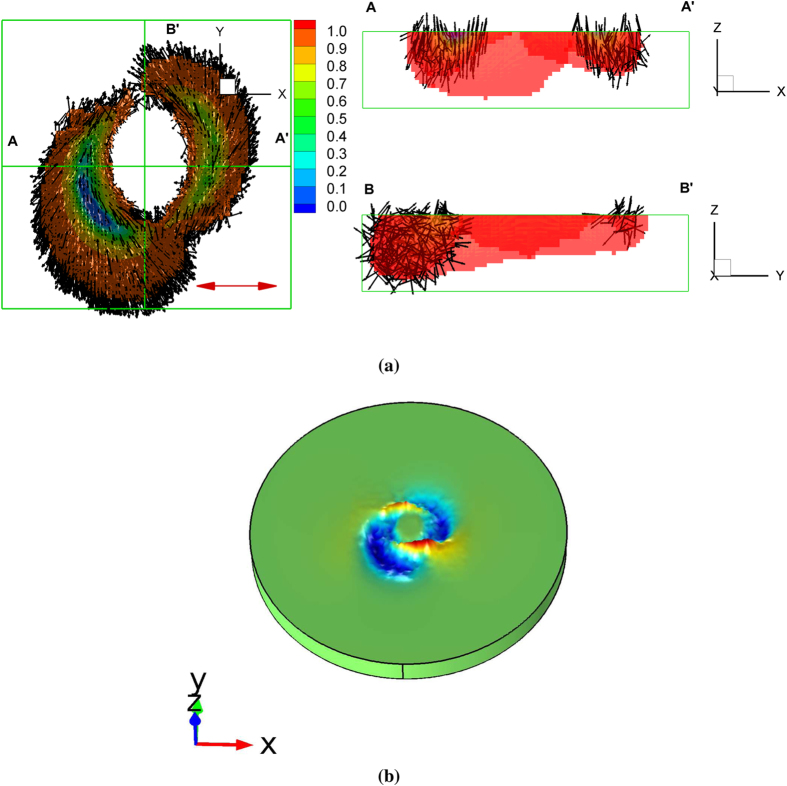
Simulation results in the case of a linearly polarized vortex laser beam. (**a**) The spatial distribution of the *trans* vector (The color denotes the magnitude of the *trans* vector, 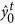. The region of 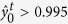 where the photoisomerization is negligible was filtered out). (**b**) Shape deformation (A color denotes the variation in the *z* direction). The positive vortex topological charge ξ = +10 is used.

Figure 7 illustrates the spatial distribution of the *trans* vector due to the illumination of the linearly polarized vortex laser beam when ξ = +10 and the resultant surface deformation of the polymer. This is the case where the deformation is relatively large and clearly observed experimentally^15^. Figure 7(a) illustrates the *trans* vectors superimposed on the time averaged magnitude of the *trans* state. The results illustrate where the light-induced *trans-cis-trans* isomerization of the azobenzene most strongly occurs. The *trans* vectors are found to be approximately aligned with the rotation direction of the vortex field. Note also the reduction of the *trans* order in regions associated with larger light intensity. These regions contain a larger concentration of the randomly ordered *cis* as illustrated in the supplementary text. Such effects contribute to the deformation as the *trans* vector order parameter in (5) is reduced. Photoisomerization becomes negligible at the core of the vortex for higher topological charge as expected due to a lack of an optical field at the beam core. Along the beam’s perimeter, the microstructure evolves asymmetrically due to the light intensity and phase induced by the topological charge. This case is more complicated than linearly or circularly polarized light since the vortex beam contains electric field components in all three Cartesian directions as it propagates in the *z* direction^48^. Figure 7(b) illustrates the resultant surface relief pattern. The double-arm spiral structure pattern in the clockwise (or left-handed) direction due to the positive ξ is clearly observed. Although not shown, we also verify that the direction of surface relief changes to the counter-clockwise direction if ξ = −10 (see the supplemental material).

**Figure 8:**
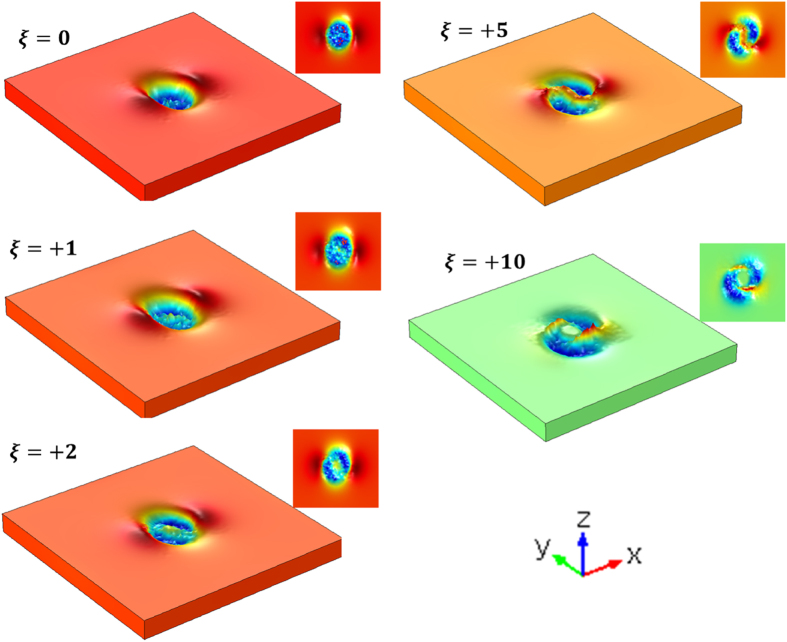
The dependence of surface relief upon the topological charge, ξ.

Figure 8 represents the surface relief patterns induced by a linearly polarized vortex beam with the different topological charge. This figure reveals that the numerical results are qualitatively in good agreement with experiments^15^. In the case of ξ = 0, two lobes along the *x* direction occur as observed in a linearly polarized Gaussian beam case. As ξ increases, these lobes rotate in the clockwise direction and the spiral structure having two-arms are produced for a high ξ. Experiments given in the literature indicate that the spiral surface relief structures induced by the vortex beam are strongly influenced by the vortex topological charge and the wavefront handedness. Here, we illustrate that this behavior is strongly dependent on the microstructure of azobenzene within the polymer network as it responds to the spatial optical intensity distribution of the light as well as its phase and handedness.

## Discussion

In summary, we have found that dipole forces strongly influence the evolution of *trans* and *cis* azobenzene states within monodomain and polydomain polymer networks. In addition, the coupling between the polymer network and azobenzene leads to significantly different photostrictive deformation. This was validated against linear polarized light applied homogeneously to a polymer surface and against surface relief grating structures that are excited with circularly polarized light and optical vortex beams with different topological charge. Although not shown, we also confirm similar surface relief grating deformation for the case of linear polarized light with respect to data in the literature^20^. Model prediction of the observed behavior requires implementation of Maxwell’s time dependent equations, linear momentum, and two electronic structure equations governing the evolution of the azobenzene *trans* and *cis* states. Interactions among the material constituents with light are contained within the local charge density of the azobenzene and the photostrictive parameters that couple azobenzene to the polymer network. This behavior is complicated by competing mechanisms associated with *trans-cis-trans* and *trans-cis* photoisomerization. A reduction of order due to the formation of the *cis* state leads to contraction of deformation to an isotropic state relative to the initial *trans* configuration. Simultaneously, reorientation of the *trans* vector is found to play an important role in the deformation in regions of smaller *cis* concentration. Importantly, we find that the photostrictive parameters are opposite in sign when simulating free bending of films versus surface relief grating deformation. It is suggested that this may be due to the underlying monomer structure of the azobenzene and its spacer length which may influence macroscopic deformation in significantly different ways. The model and comparisons with data suggest long spacers exhibit prolate liquid crystal polymer deformation while short spacers exhibit oblate behavior.

## Methods

The dynamics of electronic displacement and EM waves are implemented in three dimensions and solved using the finite difference time domain (FDTD) method proposed by Yee^49^ of second-order accurate in both space and time. For homogeneously applied UV light, a representative volume element (RVE) of a width equal to the wavelength of the applied electric field, *λ*_0_ = 370 nm, is considered as illustrated in Fig. 1(a) in the supplemental text, which satisfies periodic boundary conditions along the *x* and *y* directions. The azobenzene material is 13.5*λ*_0_ long, which is about 1/3 to 1/2 the thickness of a typical free standing film^31^,^40^. Perfectly matched layers (PML) are applied on the top and bottom to minimize wave reflection from the boundaries. A linearly polarized electric field of a plane wave is applied on the top. The computational mesh sizes used for the electromagnetic wave propagation region and for the azo-polymer are 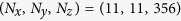 and 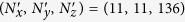, respectively.

Figure 1(b) in the supplemental text represents the computational domain used to simulate and analyze surface texture due to the *trans-cis-trans* photoisomerization. The azobenzene polymer lies in the middle of the domain and is surrounded by a vacuum where the electric field source initiates and propagates into the material. It has a volume 2*λ*_0_ long in the *z* direction and 8*λ*_0_ wide in both *x* and *y* directions. Perfectly matched layers (PML) are applied on every boundary surface to minimize wave reflection from the boundaries. Several types of polarized laser beams are applied on the top: linearly and circularly polarized Gaussian laser beams and linearly polarized vortex laser beams. A uniformly distributed computational mesh sizes was used for the electromagnetic wave propagation and for the azobenzene polymer using a grid size of 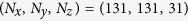 and 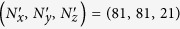 in the *x*, *y*, and *z* directions, respectively. According to the grid size, the maximum allowable normalized time step by the equation is 5.77 × 10^−2^. For numerical stability^50^, Δ*t* = 0.05 is used in all numerical simulations.

When the transient behavior of the microstructure reaches steady-state oscillations, the *trans* azobenzene microstructures are stored and inserted into a finite element model to solve Eq. (10) in the supplementay material that couples elastic behavior of the polymer network with the electronic coordinates. This is achieved by importing computational results obtained from the FDTD simulation for 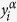 into the commercial package COMSOL to determine the stress and displacement fields. In this case, the microstructure evolution from FDTD simulations is assumed to be driven solely by the light source. Any reorientation of 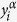 due to internal stress generation is neglected.

## Additional Information

**How to cite this article**: Bin, J. and Oates, W. S. A Unified Material Description for Light Induced Deformation in Azobenzene Polymers. *Sci. Rep.* **5**, 14654; doi: 10.1038/srep14654 (2015).

## Supplementary Material

Supplementary article
