# Supplementary material for: A Unified Material Description for Light Induced Deformation in Azobenzene Polymers
Source: Sci Rep. 2015 Oct 6;5:14654. doi: 10.1038/srep14654 (PMC4594130; doi:10.1038/srep14654)
Supplement: Supplementary article [file srep14654-s1.pdf]

Supplemental Text: A Unified Material Description for Light Induced Deformation in Azobenzene Polymers

Jonghoon Bin* and William S. Oates†

Florida Center for Advanced Aero Propulsion (FCAAP)

Department of Mechanical Engineering

Florida State University

Tallahassee, FL 32310

The complete set of governing equations describing the photo-responsive behavior of the azobenzene liquid crystal polymer network is given here to further clarify the modeling approach and elucidate the driving forces due to light induced deformation. The set of equations used to describe photomechanical deformation in the azo-LCNs is based on a Lagrangian density and dissipative potential that contains free space energy ($\mathcal{L}_F$), kinetic and stored energy of the solid ($\mathcal{L}_M$), electronic interactions ($\mathcal{L}_I$), and dissipation due to light scattering and photochemical inefficiencies ($\mathcal{D}$). These terms are given by

$$\begin{aligned}
\mathcal{L}_F &= \frac{1}{2}\left(\epsilon_0 E_i E_i - \frac{1}{\mu_0} B_i B_i\right) \\
\mathcal{L}_M &= \frac{\rho^0}{2}\dot{x}_i\dot{x}_i + \sum_\alpha \frac{m^\alpha}{2}\dot{y}_i^\alpha \dot{y}_i^\alpha - \rho^0 \Sigma(\varepsilon_{ij}, y_i^\alpha, y_{i,j}^\alpha) \\
\mathcal{L}_I &= J_i A_i - q\phi \\
\mathcal{D} &= -\sum_\alpha \frac{\gamma^\alpha}{2}\dot{y}_i^\alpha \dot{y}_i^\alpha .
\end{aligned} \tag{1}$$

The electromagnetic field Lagrangian density ($\mathcal{L}_F$) includes E_i as the electric field and B_i as the magnetic flux density, both in the spatial frame. The free space permittivity and permeability are ϵ_0 and μ_0, respectively. The interaction Lagrangian ($\mathcal{L}_I$) includes the spatial frame current density J_i, magnetic vector potential A_i, bound charge density q, and electric potential ϕ. The matter Lagrangian density per undeformed volume ($\mathcal{L}_M$) includes separate kinetic energies for the center-of-mass in the first term, a set of optical modes in the second term, and the last term is the

*Email: jbin@fsu.edu

†Email: woates@fsu.edu, Telephone: (850) 645-0139

stored energy. The kinetic energies are defined by the mass density per undeformed volume ρ^0 and velocity $\dot{x}_i$ and the electronic kinetic energy written as a function of the material time derivatives of the electronic vector order parameters, $\dot{y}_i^\alpha$, and the effective mass density with units of mass per undeformed volume, m^α. Two order parameters ($\alpha = t, c$) are considered to independently model the *trans* and *cis* states. The stored energy per undeformed volume ($\rho^0\Sigma$) in $\mathcal{L}_M$ is a function of the small strain tensor ε_{ij}, the electronic coordinate (y_i^α), and its gradient ($y_{i,j}^\alpha$)[1,2]. The dissipative energy $\mathcal{D}$ describes losses associated with light scattering and photochemical reactions in terms of the velocity of the electronic coordinates and the damping parameter γ^α.

The stored energy and the electronic coordinate vector order parameters are defined for characterizing material continuum length scales while retaining critical underlying light-matter characteristics. The model assumes that the electronic coordinates are summed and averaged over a continuum representative volume element. This formulation is non-relativistic where material velocities must be much smaller than the speed of light. Within the visible and ultra-violet light spectra, the oscillation velocity of the charged particles is still approximately three orders of magnitude smaller than the speed of light. This assumes that the charged particles do not displace more than 10% of the size of an azobenzene molecule. An important component of this formulation is the choice of the stored energy function contained within $\mathcal{L}_M$, which is expressed in the following section.

Stored energy relations

The stored energy function Σ consists of four different contributions: Σ_t and Σ_c are non-convex energy functions for the *trans* and the *cis* states, respectively; Σ_m is the elastic energy of the glassy polymer network, and Σ_{coupl} defines the coupling between the *trans* vector coordinate and the polymer network deformation. Deformation attributed to the *cis* state is neglected since this phase normally leads to disorder. Furthermore, the *trans* coordinate is reduced in magnitude as the *cis* concentration increases. The energy function is written as

$$\Sigma(\epsilon_{ij}, y_i^\alpha, y_{i,j}^\alpha) = \Sigma_t(y_i^t, y_{i,j}^t) + \Sigma_c(y_i^c, y_{i,j}^c) + \Sigma_m(\epsilon_{ij}) + \Sigma_{coupl}(\epsilon_{ij}, y_i^t) \tag{2}$$

in terms of the the small strain tensor ϵ_{ij}, the electronic vector order parameters y_i^α and their gradients $y_{i,j}^\alpha$. Each part of the stored energy density is defined by

$$
\begin{aligned}
\Sigma_t &= \frac{a^t}{2} y_i^t y_i^t + \frac{b^t(\hat{y}_0^t)}{4} y_i^t y_i^t y_j^t y_j^t + \frac{a_0^t}{2} y_{i,j}^t y_{i,j}^t \\
\Sigma_c &= \frac{a^c}{2} y_i^c y_i^c + \frac{b^c(\hat{y}_0^t)}{4} y_i^c y_i^c y_j^c y_j^c + \frac{a_0^c}{2} y_{i,j}^c y_{i,j}^c \\
\Sigma_m &= \frac{c_{ijkl}}{2} \epsilon_{ij} \epsilon_{kl} \\
\Sigma_{coupl} &= -b_{ijkl} \epsilon_{ij} y_k^t y_l^t
\end{aligned} \tag{3}
$$

where the phenomenological parameters a^α and $b^\alpha(\hat{y}_0^t)$ for $\alpha = t, c$ govern the evolution of the electronic coordinates, a_0^α is a penalty on gradients of y_i^α, b_{ijkl} is a fourth order tensor that couples the *trans* coordinate vector order parameter to stress. It should be noted that we neglect any coupling between two vector order parameters and therefore conservation of concentration of the two material states is not necessarily guaranteed. In Eq. (3), the parameters $a^\alpha < 0$ and $b^\alpha(\hat{y}_0^t) > 0$ which creates the non-convex function given in the main paper (Figure 2). The higher order parameter, b^α, is defined to be a function of a time averaged *trans* state, $\hat{y}_0^t$, to model the slower time dynamics of photoisomerization relative to dynamics that occur at visible and UV light frequencies. This equation is given in the following section. The higher order model parameter $b^\alpha(\hat{y}_0^t)$ changes during *trans-cis* photoisomerization such that the *trans* coordinate reduces in magnitude while the *cis* coordinate increases from near zero. This assumes a loss of nematic order as the concentration of the *cis* state increases. The functional forms of these phenomenological parameters are assumed to be $b^t(\hat{y}_0^t) = b_0^t/(\hat{y}_0^t)^2$ and $b^c(\hat{y}_0^t) = b_0^c/(1 - \hat{y}_0^t)^2$ where b_0^t and b_0^c are positive constants. The time averaged electronic state is restricted to $0 < \hat{y}_0^t < 1$ such that b^t and b^c are bounded and $\hat{y}_0^t = 1$ and $\hat{y}_0^t = 0$ denote the fully *trans* and the fully *cis* state, respectively. All the parameters, excluding the photostrictive coefficients, are summarized in Table 1. The photostrictive parameters are described in more detail in the subsequent section.

Governing equations

Minimization of the Lagrangian energy densities and dissipative energy function from Eq. (1) leads to the time-dependent set of electronic displacement balance equations, Maxwell's equations, and linear momentum equations. An additional auxilliary equation associated with photochemical reaction rates is also discussed.

Electronic Displacement Balance Relations

Two electronic force balance equations for the *trans* and *cis* states are obtained through energy minimization with respect to y_i^α. These equations quantify the time dependent behavior of the optically active *trans* and *cis* azobenzene states as a function of light excitation. The absorption spectra depend on the concentration of the *trans* and *cis* states. The *trans* state strongly absorbs UV light (∼370 nm) and the *cis* state strongly absorbs visible light with wavelengths in the 460 nm regime. The resulting dynamic equation is

$$m^\alpha \frac{d^2 y_i^\alpha}{dt^2} + \gamma^\alpha \frac{dy_i^\alpha}{dt} + \frac{\partial \Sigma}{\partial y_i^\alpha} = \frac{\partial}{\partial x_j}\left(\frac{\partial \Sigma}{\partial y_{i,j}^\alpha}\right) + q^\alpha E_i + \sum_\nu q^{\alpha\nu} y_j^\nu E_{i,j} + e_{ijk}\dot{x}_j \sum_\nu q^{\alpha\nu} y_l^\nu B_{k,l} \tag{4}$$

where ν is t or c for the *trans* and *cis* vector order parameter, respectively. q^α is the effective bound charge density per undeformed volume given by equation Eq. (1) in the main paper and $q^{\alpha\nu}$ is the charge density associated with the higher order gradients on the electric field and magnetic flux density. The higher order terms were identified to be negligible and is therefore not used in the current study.

The time-averaged magnitude of the *trans* state, $\hat{y}_0^t$, which is approximately $\left[(\bar{y}_1^t)^2 + (\bar{y}_2^t)^2 + (\bar{y}_3^t)^2\right]^{1/2}$ at a given time where the overbar represents time-average[3, 4], is treated on the time scale of photoisomerization which is significantly slower than the optical wavelength time period. The time-averaged magnitude of the *trans* state is determined by

$$\frac{d\hat{y}_0^t}{dt} + \frac{1}{\tau_{avg}}\hat{y}_0^t = \frac{1}{\tau_{avg}}\left[1 - \chi\alpha(\mathbf{e}\cdot\mathbf{E})^2\right] \tag{5}$$

where $\alpha = \dfrac{|\mathbf{y}^t \cdot \mathbf{e}|}{|\mathbf{y}^t||\mathbf{e}|}$ and $\mathbf{e}$ is the unit vector representing the direction of light polarization. χ is a sensitivity parameter governing the amount of photoisomerization lying on the range of $0 \le \chi < 1/(\mathbf{e}\cdot\mathbf{E})^2$. For example, if $\chi = 0$, the time averaged *trans* state is always one (zero photoisomerization). As χ increases, the time averaged *trans* state will be reduced if the electronic oscillation increases at a particular light wavelength. The time constant τ_{avg} is defined to be on the order of the photoisomerization rate (∼10 ps). This equation must be solved simultaneously with all other governing equations to determine the evolution of the azobenzene state.

Maxwell's equations

Minimization of the field quantities leads to the Maxwell equations in the spatial configuration given by

$$\begin{aligned}
&e_{ijk}E_{k,j} = -\frac{\partial B_i}{\partial t} \\
&e_{ijk}B_{k,j} = \mu_0 \left(\epsilon_0 \frac{\partial E_i}{\partial t} + J_i \right) \\
&\epsilon_0 E_{i,i} = q \\
&B_{i,i} = 0.
\end{aligned} \tag{6}$$

For the azo-LCN model, both the surface current and surface charge are set to zero and the charge density is

$$q = -P_{i,i} + Q_{ij,ij} \tag{7}$$

where P_i and Q_{ij} are the polarization and the quadrupole density, respectively. The polarization and the quadrupole are determined from the internal electronic coordinates using

$$\begin{aligned}
P_i &= \sum_{\nu} q^{\nu} y_i^{\nu} \\
Q_{ij} &= \frac{1}{2} \sum_{\nu,\mu} q^{\nu\mu} y_i^{\nu} y_j^{\mu}
\end{aligned} \tag{8}$$

for $\mu, \nu = t, c$ which is valid in the small strain limit.

The current density, neglecting magnetization effects, is

$$J_i(\mathbf{x}, t) = \frac{\partial P_i}{\partial t} + (P_i \dot{x}_j)_{,j} - (P_j \dot{x}_i)_{,i} - \frac{\partial Q_{ji,j}}{\partial t} - (Q_{ij}\dot{x}_k)_{,jk} + (Q_{jk}\dot{x}_i)_{,jk}. \tag{9}$$

Linear momentum equations

We assume linear elastic behavior of a glassy polymer coupled to quadratic dependence on the *trans* vector order parameter. Due to large disparity in time scales between optical waves and elastic waves and viscoelasticity within glassy polymer, we neglect the rate dependent deformation and solve the quasi-static form of linear momentum given by

$$\sigma_{ij,j} = (c_{ijkl}\epsilon_{kl} - b_{ijkl}y_k^t y_l^t - \sigma_{ij}^R)_{,j} = 0 \tag{10}$$

where the reference stress is $\sigma_{ij}^R = -b_{ijkl}y_k^{t0}y_l^{t0}$ and the superscript $t0$ denotes the initial microstructure configuration of the *trans* state. The sign of the photostrictive parameters determine elongation or contraction with respect to the *trans* vector order parameter. The two cases considered in the simulations uses the parameter values in Table 2. In this case, the coupling tensor b_{ijkl} reduces to $b_{ijkl} = 0$ excluding $b_{1111} = b_{2222} = b_{3333} = b_1$, $b_{1122} = b_{1133} = b_{2233} = b_2$, and $b_{1212} = b_{1313} = b_{2323} = b_3$. Also note due to symmetry that $b_{ijkl} = b_{klij}$ and $b_{ijkl} = b_{jikl} = b_{ijlk}$. Similar symmetry exists for the elastic tensor c_{ijkl}.

The total Cauchy stress denoted by σ_{ij} contains components associated with deformation and the internal electronic order parameters. Assuming an isotropic elastic medium, the expanded form of the Cauchy stress is[5]

$$\begin{aligned}
\sigma_{11} &= c_{1111}\epsilon_{11} + c_{1122}(\epsilon_{22} + \epsilon_{33}) - b_1 y_1^t y_1^t - b_2(y_2^t y_2^t + y_3^t y_3^t) - \sigma_{11}^R \\
\sigma_{22} &= c_{1111}\epsilon_{22} + c_{1122}(\epsilon_{11} + \epsilon_{33}) - b_1 y_2^t y_2^t - b_2(y_1^t y_1^t + y_3^t y_3^t) - \sigma_{22}^R \\
\sigma_{33} &= c_{1111}\epsilon_{33} + c_{1122}(\epsilon_{11} + \epsilon_{22}) - b_1 y_3^t y_3^t - b_2(y_1^t y_1^t + y_2^t y_2^t) - \sigma_{33}^R \\
\sigma_{12} &= c_{1212}\epsilon_{12} - b_3 y_1^t y_2^t - \sigma_{12}^R \\
\sigma_{13} &= c_{1212}\epsilon_{13} - b_3 y_1^t y_3^t - \sigma_{13}^R \\
\sigma_{23} &= c_{1212}\epsilon_{23} - b_3 y_2^t y_3^t - \sigma_{23}^R
\end{aligned} \tag{11}$$

where $c_{1111} = \frac{1-\vartheta}{(1+\vartheta)(1-2\vartheta)}Y$, $c_{1122} = \frac{\vartheta Y}{(1+\vartheta)(1-2\vartheta)}$, $c_{1212} = \frac{Y}{(1+\vartheta)}$, $Y = 1\times10^8$ N/m^2 is Young's modulus, and $\vartheta = 0.45$ is Poisson's ratio. Table 2 represents parameters used in the present study. An uniform bending of a monodomain thin film during the *trans-cis* photoisomerization is numerically performed using photostrictive parameters that assume long spacers otherwise surface relief deformation during the *trans-cis-trans* photoisomerization are performed using that in the case of short spacers.

Supplemental Computational Analysis

The computational domain for the two configurations simulated in the main text is shown in Figure 1. The domain in Figure 1(a) is used to simulate bending from uniform light exposure while Figure 1(b) describes the computational domain used in the surface relief grating simulations.

In the visible light regime (λ_0= 442 nm), the *trans-cis-trans* photoisomerization leads to anisotropic evolution of the azobenzene microstructure as the *trans* state molecules reorient perpendicular to the electric field direction of the polarized light. During this process there is also an amount of concentration build-up of the *cis* state during to *trans-cis* photoisomerization. As illustrated in the main text, this behavior is particularly complex for the case of a vortex beam. Supplementary plots of simulations of the surface relief grating are given to clarify the model predictions leading to surface deformation shown in the main article. In Figure 2, the formation of the *cis* state from circularly polarized light is shown. This illustrates a complex competition between order-disorder (*trans-cis*

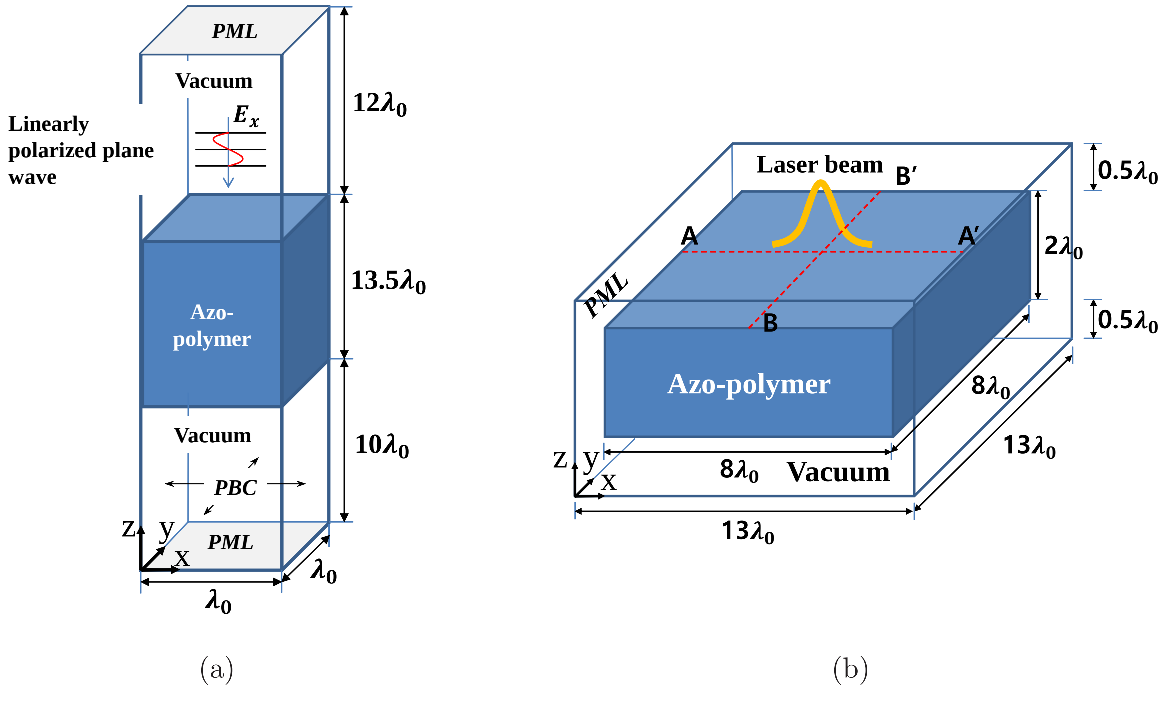

Figure 1: Schematic of the azobenzene layer sandwiched between two vacuum layers. (a) Uniform exposure of linearly polarized light. (b) Light beam geometry for linearly and circularly polarized light and vortex beams with different topological charge. PML stands for perfectly matched layers.

photoisomerization) near the center of the beam and order-disorder-reorder (*trans-cis-trans* photoisomerization) near the perimeter of the beam. The disorder near the beam center leads to reduction in deformation and the dimple seen in Figure 6 in the main article. The protrusions around the perimeter are attributed to reorientation of the *trans* order parameter in regions of minimal *cis* concentration. Similar effects, yet more complicated, are seen in the case of a vortex beam as shown in Figure 3. In this case, we also see the formation of the *cis* state thus illustrating the interactions between *trans* reorientation and order-disorder behavior from formation of *cis* during visible light excitation.

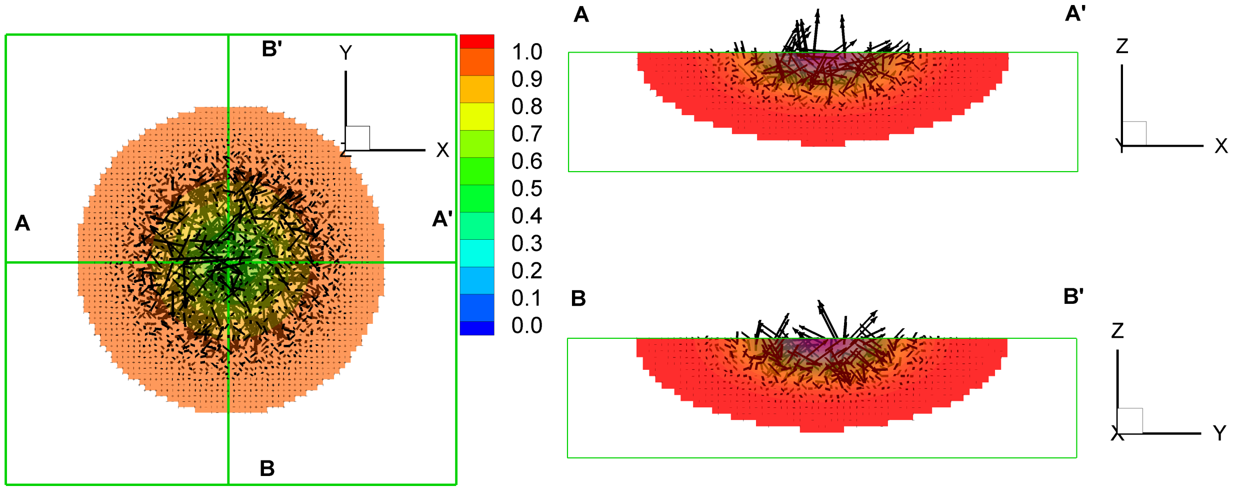

Figure 2: Illustration of the formation of the randomly ordered *cis* state during visible light excitation from a circularly polarized laser beam. This microstructure corresponds to the simulation illustrated in Figure 6 in the main article. The color bar corresponds to the magnitude of the *trans* vector for comparisons to the *cis* state.

Due to the complexity of the light intensity and phase induced by the topological charge in the case of surface deformation due to the exposure of a linearly polarized vortex laser beam, we also compare the microstructure evolution to the root-mean-squared (RMS) electric field as shown in Figure 4. From this figure, it is clearly observed that besides the transverse optical field components E_x and E_y, the longitudinal component E_z is also produced but E_z is smaller by a factor $1/k\omega$

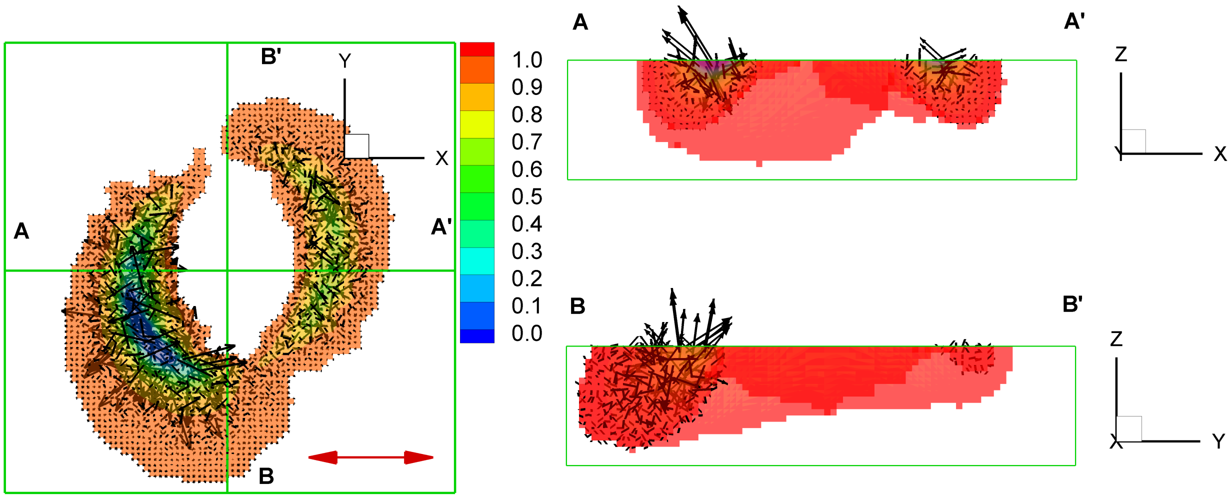

Figure 3: Illustration of the formation of the *cis* state during visible light excitation from a vortex beam with topological charge, $\xi = +10$. This microstructure corresponds to the simulation illustrated in Figure 7 in the main article. The color bar corresponds to the magnitude of the *trans* vector for comparisons to the *cis* state.

compared to the dominant transverse optical field component, E_x[6]. The intensity of E_z finally leads to the two-armed spiral struture as shown in Figure 7(b) of the main paper.

We further analyze the model by switching the sign of the topological charge to be $\xi = -10$ (see Figure 5). For this case, it is evident that the helical pattern on the surface is opposite to that in Figure 8(b) in the main paper.

Table 1: A summary of the material parameters. All parameters for the trans state are given.

Symbol	Value	Units	Definition
m^μ	1×10^{-28}	kg/m^3	Mass density of *trans* and *cis* electronic coordinates
a^t	$-1.27 \times 10^{31} m^t$	N/m^4	Trans free energy parameter
a^c	$0.66a^t$	N/m^4	Cis free energy parameter
b_0^t	$-a^t/(\hat{y}_0^t)^2$	N/m^6	Trans free energy parameter
b_0^c	$-a^c/(1-\hat{y}_0^t)^2$	N/m^6	Cis free energy parameter
a^{tct}	$-2 \times 10^{-3} a^t$	N/m^4	Trans-cis-trans photoisomerization parameter
γ_0^t	6×10^{-42}	$N \cdot s/m^4$	Isotropic damping of the trans state
γ_0^c	γ_0^t	$N \cdot s/m^4$	Isotropic damping of the cis state
q_0	0.75×10^{-10}	C/m^3	Nominal charge density
N_0	1.33×10^{10}	–	Number of electronic particles within a volume element
q^t	$q_0\hat{y}_0^t$	C/m^3	Charge density of the trans state
q^c	$q_0(1-\hat{y}_0^t)$	C/m^3	Charge density of the cis state
E_0	614	V/m	Nominal applied field
a_0	1×10^{-14}	N/m^2	Diffusion parameter, $a_0 = a_0^t = a_0^c$
τ_{avg}	1×10^{-12}	s	Photoisomerization time constant

Table 2: Parameters used in the model for shape deformation due to *t*-*c*-*t* photoisomerization process.

Type	b_1	b_2	b_3
Long spacer	+0.05Y	-0.015Y	$(b_1 - b_2)/2$
Short spacer	-0.05Y	+0.015Y	$(b_1 - b_2)/2$

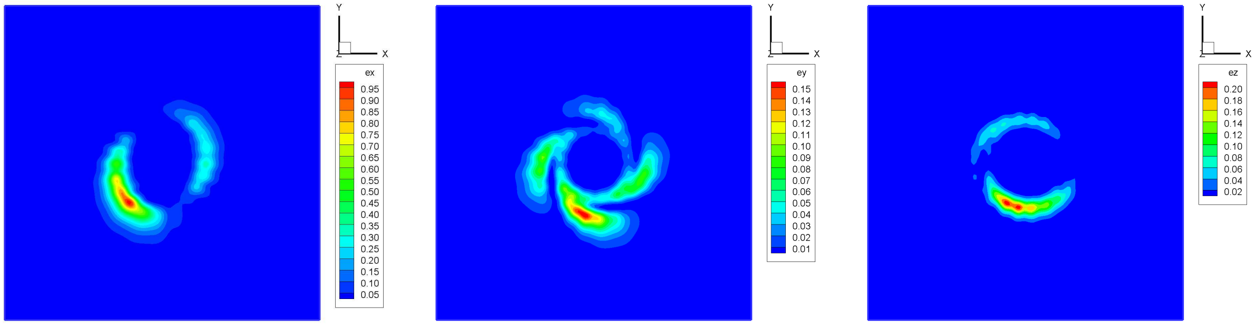

(a) **(b)** **(c)**

Figure 4: Time-averaged electric field on the xy plane at the top-plane of the plate. (a) $\bar{E}_x$. (b) $\bar{E}_y$. (c) $\bar{E}_z$

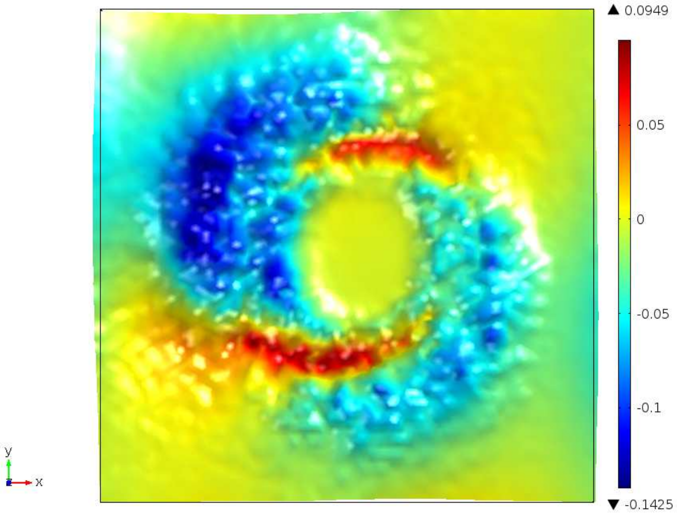

Figure 5: Deformation due to a linearly polarized vortex laser beam when the negative vortex topological charge $\xi = -10$ is used.

References

[1] L. Malvern, *Introduction to the Mechanics of a Continuous Medium.* Englewood Cliffs, NJ: Prentice-Hall, Inc., 1969.

[2] D. Nelson, *Electric, Optic, and Acoustic Interactions in Dielectrics.* New York: John Wiley and Sons, 1979.

[3] I. K. Lednev, T.-Q. Ye, R. E. Hester, and J. N. Moore, "Femtosecond time-resolved UV-Visible absorption spectroscopy of trans-azobenzene in solution," *The Journal of Physical Chemistry*, vol. 100, no. 32, pp. 13 338–13 341, 1996.

[4] T. Fujino, S. Y. Arzhantsev, and T. Tahara, "Femtosecond time-resolved fluorescence study of photoisomerization of trans-azobenzene," *The Journal of Physical Chemistry A*, vol. 105, no. 35, pp. 8123–8129, 2001.

[5] W. Zhang and K. Bhattacharya, "A computational model of ferroelectric domains. Part I: Model formulations and domsain switching," *Acta. Mater.*, vol. 53, pp. 185–198, 2005.

[6] W. L. Erikson and S. Singh, "Polarization properties of Maxwell-Gaussian laser beams," *Phys. Rev. E*, vol. 49, no. 6, pp. 5778–5786, 1994.
